# Regulation and Function of Interferon-Lambda (IFNλ) and Its Receptor in Asthma

**DOI:** 10.3389/fimmu.2021.731807

**Published:** 2021-11-10

**Authors:** Susanne Krammer, Cristina Sicorschi Gutu, Janina C. Grund, Mircea T. Chiriac, Sabine Zirlik, Susetta Finotto

**Affiliations:** ^1^ Department of Molecular Pneumology, Friedrich-Alexander-Universität (FAU) Erlangen-Nürnberg, Universitätsklinikum Erlangen, Erlangen, Germany; ^2^ Medical Clinic 1, Friedrich-Alexander-Universität (FAU) Erlangen-Nürnberg, Universitätsklinikum Erlangen, Erlangen, Germany

**Keywords:** asthma, interferon, rhinovirus, exacerbation, TLR7/8, epithelial cells

## Abstract

Asthma is a chronic respiratory disease affecting people of all ages, especially children, worldwide. Origins of asthma are suggested to be placed in early life with heterogeneous clinical presentation, severity and pathophysiology. Exacerbations of asthma disease can be triggered by many factors, including viral respiratory tract infections. Rhinovirus (RV) induced respiratory infections are the predominant cause of the common cold and also play a crucial role in asthma development and exacerbations. Rhinovirus mainly replicates in epithelial cells lining the upper and lower respiratory tract. Type III interferons, also known as interferon-lambda (IFNλ), are potent immune mediators of resolution of infectious diseases but they are known to be involved in autoimmune diseases as well. The protective role of type III IFNs in antiviral, antibacterial, antifungal and antiprotozoal functions is of major importance for our innate immune system. The IFNλ receptor (IFNλR) is expressed in selected types of cells like epithelial cells, thus orchestrating a specific immune response at the site of viruses and bacteria entry into the body. In asthma, IFNλ restricts the development of TH2 cells, which are induced in the airways of asthmatic patients. Several studies described type III IFNs as the predominant type of interferon increased after infection caused by respiratory viruses. It efficiently reduces viral replication, viral spread into the lungs and viral transmission from infected to naive individuals. Several reports showed that bronchial epithelial cells from asthmatic subjects have a deficient response of type III interferon after RV infection *ex vivo*. Toll like Receptors (TLRs) recognize pathogen-associated molecular patterns (PAMPs) expressed on infectious agents, and induce the development of antiviral and antibacterial immunity. We recently discovered that activation of TLR7/8 resulted in enhanced IFNλ receptor mRNA expression in PBMCs of healthy and asthmatic children, opening new therapeutic frontiers for rhinovirus-induced asthma. This article reviews the recent advances of the literature on the regulated expression of type III Interferons and their receptor in association with rhinovirus infection in asthmatic subjects.

## 1 Introduction: Asthma and Its Pathogenesis

Asthma is a chronic respiratory disease affecting the life of millions of people worldwide. The signs and symptoms are wheezing, cough and dyspnea. These asthmatic manifestations have a great impact on people´s life quality, especially in patients with uncontrolled asthma in low-income countries.

The pathogenesis of asthma is not completely understood as it has both genetic and environmental factors influencing the disease. Environmental triggers causing asthma development and exacerbation are allergens, air pollution, like cigarette smoke, as well as volatile organic compounds, household compounds or viral, microbial and fungal infections.

Atopy, a condition in which the immune system is more sensitive to common, otherwise not toxic substances, called allergens, is a triad of hyperreactive tissues (allergic eczema, in the skin, allergic rhinitis, in the nose and allergic asthma, in the lower airways). Allergen exposure still remains the strongest risk factor for developing allergic asthma in susceptible subjects. Therefore, the main focus of asthma research groups during the last decades has been the type 2 inflammatory responses, where T lymphocytes play a prominent role in the pathogenesis, in particular T-helper cells type 2 (TH2), their transcription factors and cytokines, e.g., Interleukin-4 (IL-4), IL-5 and IL-13 (1–5). Asthma is characterized by an imbalance between TH1 and TH2 response in the airways, following an overshooting response to a harmless antigen like allergens or pathogens. The hypersecreted cytokines IL-4, IL-5 and IL-13 lead to smooth muscle spasms, goblet cell hyperplasia and eosinophilic inflammation (6). As a result, new biological therapies such as monoclonal antibodies targeting IL-5 or IL-5 receptor were approved. Nevertheless, approximately half of asthmatics lack the type 2 phenotype, suggesting that asthma is a much more complex syndrome with many possible phenotypes. More recently, it became clear that type 2 innate lymphoid cells (ILC2) also play an important role and are main producers of IL-5 upon IL-33, IL-25 and TSLP stimulation. Additionally, T-helper cells type 17 (TH17) recruit neutrophils *via* IL-8 into the lung after antigen presentation. These neutrophils upregulate α-defensines and serine proteases thus characterizing a different manifestation of asthma than eosinophilic driven inflammation (7).

Most approaches to distinguish different asthmatic phenotypes are based on the origin and time point of the disease onset, as well as the symptom and treatment status of the patient. Siroux et al. described asthma phenotypes A-D. Group A was actively treated allergic childhood-onset asthma while B was actively treated adult-onset. Groups C (allergic) and D (non-allergic) were characterized by very mild symptoms and no need for treatment (8). Another approach was to do a cluster analysis using symptoms and eosinophilic inflammation as key parameters. Similarly, a common phenotype of early-onset atopic asthma was identified. In contrast to this type, there is a predominant eosinophilic inflammation phenotype with late onset of the disease. On the other hand the authors describe two symptom driven phenotypes, the early symptom predominant and the obese non-eosinophilic type (9). Especially patients with higher body mass index (BMI) and more eosinophilic inflammation were at risk risk of suffering more frequently from asthma exacerbations (10).

As respiratory virus infections are seen as one of many triggers for non-allergic asthma, the defense against those pathogens impacts asthma development as well. Depending on the different serotypes, rhinovirus shows a cytopathic effect on the bronchial epithelial cells. This cytotoxic effect could play an important role in increasing the susceptibility to asthma development (11). A large cohort study found an interaction between single nucleotide polymorphisms (SNPs) in the 17q21 locus, rhinovirus induced wheezing illness in childhood and asthma development (12). Despite these findings, it is still controversial whether RV infections are the cause or also a result of susceptibility to allergens and pathogens of asthmatic subjects.

Nevertheless, many asthmatics suffer from virus induced worsening of their disease, so called asthma exacerbations. These exacerbations can occur frequently and are defined as acute or subacute episodes of progressive worsening of symptoms as well as lung function. Mostly, upper and lower respiratory tract infections are responsible for these exacerbations. Characteristically, rhinovirus (RV) is considered to be the main trigger of asthmatic exacerbations, being detected in up to 70-80% of children and adult exacerbations (13–16).

Thus, it is crucial for asthmatic patients that their body produces antiviral proteins commonly known as interferons. These agents consist of three subfamilies called interferons type I, II and III. In this review, we focus on the type III interferon family also known as interferon-lambda 1-4. So far, the differential role of IFNs is not well understood although understanding their regulation might be an important target for therapeutic antiviral strategies. The current SARS-CoV-2 pandemic demonstrates even more how dangerous new virus mutations are and how essential a sufficiently controlled antiviral immune response is (17).

Here we will further discuss the role of RV infections as common triggers of asthmatic exacerbations and how type III IFNs affect them (18). Furthermore, in this narrative review, we will emphasize on the regulation and variability in the expression of interferon-lambda family members and the signaling *via* its receptor interferon-lambda receptor (IFNλR) (19).

## 2 Rhinovirus Infection and Antiviral Immune Response

The human rhinovirus (HRV) is a positive-sense single-stranded RNA [(+)ssRNA] virus belonging to the genus enterovirus of the picornaviridae family. Rhinoviruses are commonly known as respiratory viruses associated with infections of the upper respiratory tract and represent the main agent causing the common cold (20). Studies in recent years have additionally shown that HRV can trigger or promote lower respiratory tract diseases such as asthma exacerbations or other inflammatory lung diseases (21, 22).

**Figure 1:**
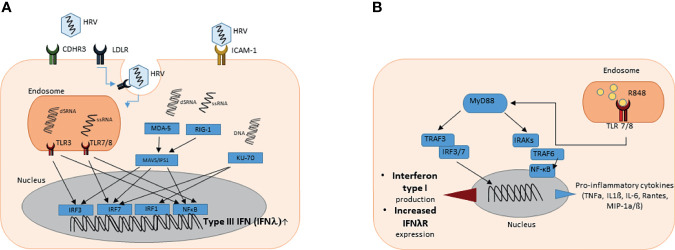
**(A)** Graphical illustration of the induction of type III interferon gene expression upon rhinovirus infection. HRV enters the cell *via* ICAM-1 receptor, CDHR3 receptor or *via* clathrin-mediated LDLR endocytosis. Viral cytosolic and endosomal ssRNA and dsRNA can be detected by PRR and PRM such as RIG-1 or MDA-5 (cytosolic) or TLR3 and TLR7/8 (endosomal), respectively. PRR and PRM induce downstream signaling cascades leading to recruitment of IRFs and NF-κB that promote type III interferon gene expression in the nucleus. **(B)** Graphical illustration of the signaling pathway after TLR 7 and 8 activation by resiquimod (R848). TLR 7 and 8 are located in intracellular vesicles, where they bind ssRNA or substances that mimic their structure. After activation, the signal is transduced *via* Myeloid differentiation primary response 88 (MyD88). Important mediators are TNF receptor-associated factor (TRAF) 3 and 6 as well as different interleukin-1 receptor associated kinase family members (IRAKs). Interferon regulatory factors (IRF) 3 and 7 and nuclear factor ‘kappa-light-chain-enhancer’ of activated B-cells (NF-κB) are the key regulatory factors that influence the gene expression in the nucleus. As a result the production of pro-inflammatory cytokines and type I interferons is enhanced.

Three species of HRV have been reported: RV group A (RV-A), RV group B (RV-B) and the recently discovered RV group C (RV-C). Together they comprise more than 100 serotypes that can be characterized by their receptor binding site (23). The majority of RV-A and RV-B, approximately 90% (major receptor group), enters the cell *via* binding intercellular adhesion molecule 1 (ICAM-1) (24). Attachment to ICAM-1 receptor leads to conformational change of the virus capsid and uncoating so that the viral genome can be released into the cytosol. The minority of HRV (minor receptor group) utilizes the low-density lipoprotein receptor (LDLR) for entering the host cell. Virus-attachment to the LDLR results in clathrin-dependent endocytosis and RNA-release into the endosome. After internalization of the receptor ligand complex by clathrin-mediated endocytosis, the internal ribosomal entry site (IRES) recruits the host translation machinery, including ribosomes, to initiate viral replication and protein biosynthesis (**Figure 1A**). In contrast, Cadherin related family member 3 (CDHR3) was recently described as a possible receptor for RV group C but little is known and this group needs further investigations (24).

Endosomal viral ssRNA can be recognized by the host *via* toll-like receptor (TLR) 7 and 8. During the viral replication process, double-stranded RNA (dsRNA) is produced. Viral dsRNA in the endosome can be detected by the host *via* the pattern recognition receptor (PRR) toll-like receptor 3 (TLR3) (25, 26). Cytosolic viral RNA can be recognized by retinoic acid-inducible gene 1 (RIG-1) or melanoma differentiation-associated gene 5 (MDA-5). Host detection of viral RNA by these PRR and pattern recognition molecules (PRM) consequently induces downstream signaling cascades. MDA5 interacts with the mitochondrial antiviral signaling proteins (MAVS) leading to recruitment of interferon regulatory factors (IRFs) 3 and 7 into the nucleus, binding to the promotor of INFλ and resulting in induction of the IFNλ family gene expression (27–29). This signaling pathway not only induces type III interferon production but also leads to higher expression of type I interferons (30). Downstream of TLR 7/8 and TLR3, the Myeloid differentiation primary response 88 (Myd88) and TIR-domain-containing adapter-inducing interferon-β (TRIF) cascades are activated and lead to a recruitment of IRFs as well as nuclear factor ‘kappa-light-chain-enhancer’ of activated B-cells (NF-κB) into the nucleus enhancing type III interferon expression (31). Notably, the combined activation of both IRFs and NF-κB was reported to be required for maximal IFNλ gene expression (32). While these pathways similarly induce type I and type III IFNs, KU-70, known as cytosolic DNA sensor, was found to rather induce type III interferon gene expression (33). The downstream mediator of KU-70 dependent IFN type III production was recently identified to be STING (Stimulator of IFN genes), which is also important in IFN type I signaling (34).

Except interferons, the pathogenesis of HRV infection involves various different cell types and cytokines as well as chemokines. Besides a direct effect on the airway epithelial cells that has been described, RV is causing a destruction of the tight junctions (35, 36). The infected epithelial cells and the neighbouring epithelium produce different cytokines and chemokines like IL-8, IP-10, G-CSF and RANTES (37). These mediators can also trigger an aggressive pro-inflammatory immune response that damages the epithelium. Additionally, it was demonstrated that TH2 cytokine driven mucus cell metaplasia reduced the virus load in human bronchial epithelial cells. The main target of the rhinovirus are the ciliated cells of the epithelium. Their infection leads to an even higher impairment of ciliary transport in asthmatics and a worsened clearance of mucus in the lung (38, 39). The antiviral immune response triggered by HRV can be divided further into an innate and an adaptive immune response. Aab et al. reported that *in vitro* B cell infection by HRV resulted in B cell proliferation, generation of infectious virions and the elicitation of pro-inflammatory cytokine production (40). Further, replication of HRV in human macrophages was shown to induce pro-inflammatory cytokine production *via* upregulation of NF-κB (41). Rajput et al. recently investigated the differences of RV-A1B and RV-C15 infections in asthmatic mice. They discovered higher expression of TH2 mediators (IL-5, IL-13 and CCL24), as well as higher peribronchial levels of IL-25, IL-33 and TSLP which are key drivers of ILC2 differentiation. As expected these effects resulted in higher numbers of ILC2 (42). This new insight supports the hypothesis that different RV subtypes show varying effects on the host and affect asthmatic patients more or less strongly.

## 3 Impact of Type III Interferon Secretion on the Respiratory Tract

The discovery of type III Interferons known as IFN-lambda in 2003 revised our knowledge about viral defense and led to a better understanding of the innate and adaptive immune response. So far, many studies and reports revealed the antiviral, anti-proliferative and immunomodulatory properties of type III IFN (27, 43–45). IFNλ family consist of four members (IFNλ 1-4) in humans and binds to the heterodimeric IFNλ receptor. IFNλ was shown to be predominantly produced upon infection at the site of entry in the epithelium of the lung or gut (46). Odendall et al. suggest that upregulation of peroxisomes during cell differentiation is a possible mechanism of preferential IFNλ production over IFNα in epithelial cells. RLRs drive the activation of IFNλ but not IFNα production *via* peroxisomal MAVS (47, 48). After the first contact of the pathogen with the epithelium the dendritic cells encounter antigen and rapidly produce IFNα as well as IFNλ (49). In the lung IFNλ is induced rapidly upon infection to control virus replication without activating inflammation. Subsequently IFNα levels increase and support immune defense while also inducing a pro-inflammatory response (50). Furthermore, there is evidence for a cross-talk between the type I and type III IFNs. Addition of IFNλ to human PBMC plus hepatoma cells co-culture increased the production of IFN type I and II (51). Similarly, pDC were also activated to produce IFNα upon IFNλ stimulation, as they highly express IFNλR.

In asthma, type III Interferons have been reported to decrease IL-4 and IL-5 production, as well as IL-13 production by T cells (3, 44, 52) providing evidence for a protective role in asthma exacerbations.

**Figure 2:**
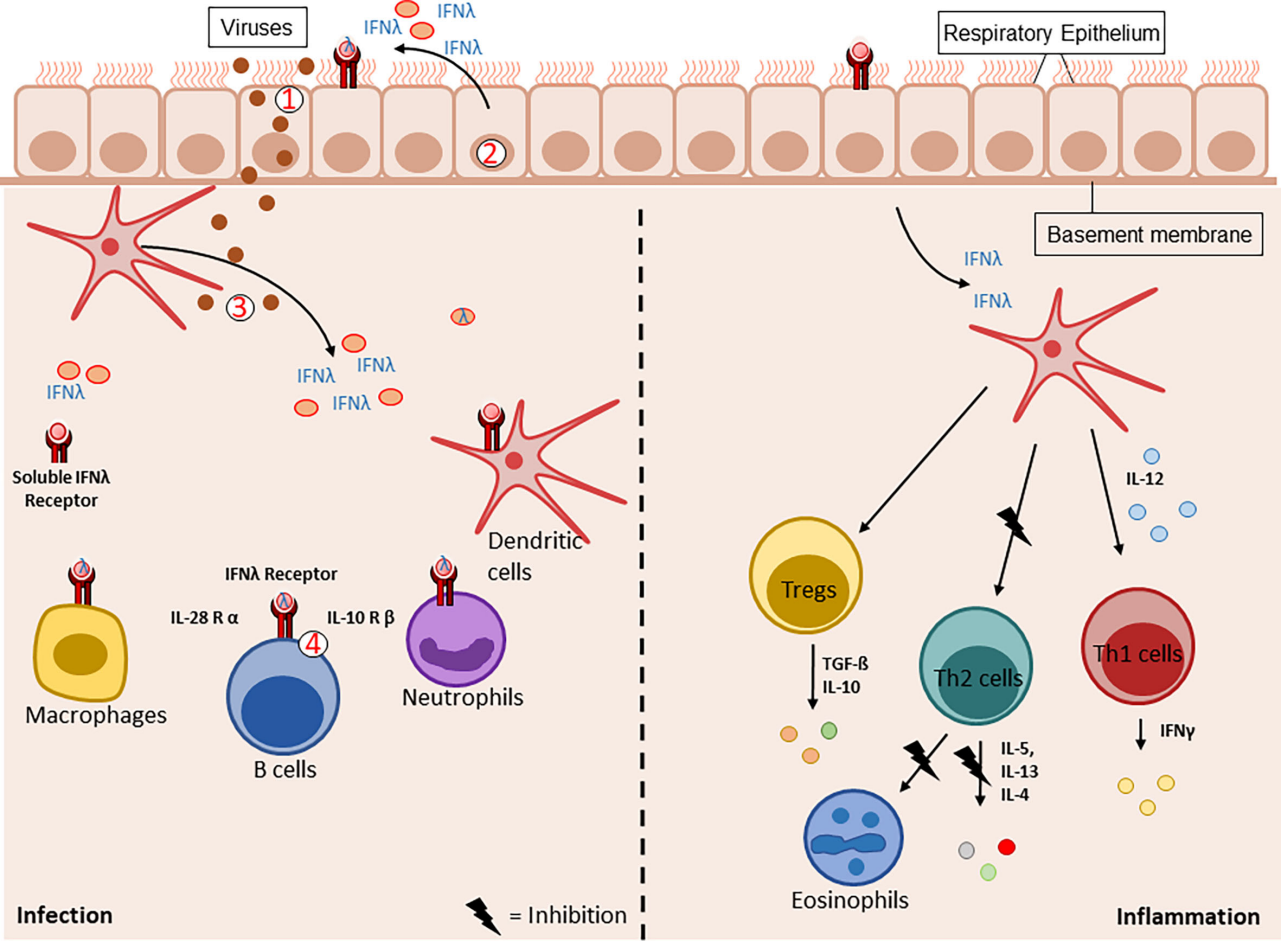
Graphical illustration of the IFNλ family members production and release during virus infection (left) vs inflammation (right). Upon viral stimulation (1), the epithelial cells (2) and dendritic cells (3) are the main producers of type III interferons. The epithelial cell barrier therefore is the first defense line against viral infections. After the release of type III interferons, they bind to their specific receptor the IFNλR (4). This receptor is expressed on different cell types like macrophages, dendritic cells, neutrophils, B-cells and epithelial cells. During inflammatory processes, IFNλ activates dendritic cells to produce IL-12. This activates the Th1 immune response and thereby suppresses Th2 mediated inflammation like recruitment of eosinophils to the site of inflammation. Under these conditions, the Treg function is maintained and supports the lung homeostasis by resolving the airway inflammation.

We and other groups demonstrated the protective role of IFN type III in experimental allergic airway disease (53). Here, balb/c wild-type mice were sensitized and challenged with ovalbumin (OVA) and additionally treated with recombinant IFNλ2 (IL-28A) or PBS intranasally. IL-28A-treated asthmatic mice had less granulocytes in the bronchoalveolar lavage (BAL), less inflammation in histological sections but higher IFNγ expression in OVA-restimulated cells isolated from the mediastinal lymph-node cells. Consistently, IFNLR1 KO asthmatic mice had higher IgE-levels, more granulocytes in the BAL and higher effector T cell responses in OVA-restimulated cells from the mediastinal lymphnode. Furthermore, this study demonstrated an immune shift from TH2/TH17 immune response to TH1 immune response following IL-28A application. The TH1 response was predominantly mediated by conventional dendritic cells *via* IFNγ (**Figure 2**, right-handside) (53). In a murine rheumatoid arthritis model, IFN λ2 treatment lead to reduction of *IL1β*, *IL17* and *IL23* mRNA expression in the joint. The numbers of neutrophils, TH17 and γδ T cells in the joint were similarly decreased. The data shows a limitation of neutrophil migration to the joint by IFNλ2 treatment (54). Another group also showed that IFNλ1 modulates TH1 and TH2 responses in a dose dependent manner. IL-13 secretion was markedly reduced while IFNγ could be induced by high doses only. Moreover, pretreatment of mDC with IFNλ1 prevented the activation of IL-13 producing T cells (55). This modulating effect on T cells was not only restricted to cytokine production (56). IFNLR1 KO mice showed a deficiency in memory T cells during influenza-A virus (IAV) infection. This effect is due to an altered migratory and antigen presentation ability of CD103+ DC (57). Both, IFNλ1 and IFNλ4, even have synergistic effects with low dose TCR-mediated stimulation and were able to enhance IFNγ production by CD8+ T cells (58). Further, peripheral DC stimulated with IFNλ upregulated CD80 and ICOS-L expression and, in combination with IFNα, an even stronger enhancement of CD80, CD83 and ICOS-L was observed (59).

These findings show that IFN type III not only modulates the T cell response directly but also indirectly *via* dendritic cells. During viral infections, IFNλ shows direct effects on epithelial cells (**Figure 2**, left handside). Remarkably, it also alters the immune response indirectly *via* the IFNλ-TSLP-axis. Thymic stromal lymphopoietin (TSLP), a regulator of adaptive immunity produced by epithelial cells was found to be induced by type III interferons after infecting mice with a live-attenuated influenza virus. TSLP stimulated migratory dendritic cells and boosted antigen-dependent germinal center reactions in draining lymph nodes. Furthermore, this resulted in higher numbers of virus-specific CD8+ T cells and an enhanced production of IgG1 and IgA. This study demonstrates a clear modulation of the adaptive immune system *via* type III interferons (60).

It was generally an assumed concept that subjects with underlying lung pathological conditions like asthma have a deficient innate immune response to infections, with significant downregulation of type I and III interferon genes (61–63). The role of type III IFNs in viral infections has been largely studied, unfortunately with mixed results so far. In the following section we discuss the results on IFNλ production in asthmatic patients in the lung during acute exacerbation or steady state asthma.

### 3.1 Role of IFN Type III in the Airways in Asthma

#### 3.1.1 Role of IFN Type III During Asthma Exacerbations

In adult cohorts, some reports found that bronchial epithelial cells from asthmatic subjects had no sufficient induction of type III interferon expression upon *in vivo* viral infection. Higher levels of IFNλ were associated with lower numbers of eosinophils in sputum, lower levels of IL-8 and less virus load in BAL. This could mean an increased susceptibility of asthmatic patients to viral infections and an impaired defense against them (62). Supporting this hypothesis, an adult asthma cohort compared levels of interferons with and without active exacerbation 4 days after the onset of symptoms. IFNα and IFNγ in the sputum were increased while IFNλ was not significantly regulated (64).

Opposed to these findings, a strong interferon response in asthmatics during acute exacerbations was found in the study published by Hansel et al. where Twenty-eight asthmatics and eleven healthy controls underwent nasal inoculation with RV16. The authors performed nasosorption during days 0, 2, 3, 4, 5 and 7 after infection. Patients had a bronchoscopy 14 days prior to RV inoculation and on day 4 afterwards. Thirty-four cytokines were measured in the nasal fluid. Here the authors detected a strong increase in interferon type II (IFNγ) and type III (IFNλ) levels in the upper airways of asthmatic patients compared to controls around the time point in which the RV infection reached its highest level in the upper airways (65). In a recent study, Veerati et al. found that viral exacerbations in patients with lung conditions, such as asthma and chronic obstructive pulmonary disease (COPD), relate to delayed rather than deficient expression of epithelial cell innate antiviral genes. Bronchial epithelial cells were obtained from healthy, severe asthmatic or COPD diagnosed individuals. Gene expression patterns were similar in all patient groups, but the kinetics of induction were delayed in the samples obtained from patients with asthma or COPD. Induced gene expression peaked at 48 h post-infection in healthy subjects. In contrast, in cells from asthmatic and COPD donors the induction was maximal at or beyond 72-96 h post infection. The authors suggested that the varied methods used in other studies might explain the discrepancies. However, once the response is initiated, it is robust and does not differ from healthy controls (66).

In paediatric studies the immune response after rhinovirus infection in asthmatic children was weak and reduced, irrespective of their atopic condition. The group included in this study consisted of 47 children who underwent bronchoscopy. After *ex vivo* infection of their bronchial epithelial cells with type 16 rhinovirus, Interferon-lambda levels could be detected. The authors analyzed the IFNβ and IFNλ protein levels 48 hours after infection. RV16 infection resulted in significant increased IFNβ protein production in all groups but the levels were significantly lower in atopic asthmatic, non-atopic asthmatic, and atopic non-asthmatic children. This reduction in IFNλ and IFNβ mRNA levels in asthmatics correlated with a significant increase in RV16 vRNA levels in both atopic and non-atopic asthmatic children when compared with those seen in healthy children. Further, IFNλ induction showed inverse correlation between epithelial damage and the airway TH2 cell profile (67). Similarly, in the study by Edwards et al., severe asthmatic children had a deficient interferon-beta and interferon-lambda induction in bronchial epithelial cell culture *ex vivo* after rhinovirus infection. In accordance to the impaired interferon response, the viral load was found significantly increased. These findings suggest the impaired innate immune response to respiratory virus could be interpreted as a characteristic of severe therapy resistant asthma (68). In addition, more than 400 children with asthma and upper respiratory symptoms, with or without wheezing, were prospectively studied in the article published by Miller et al. (69). Human rhinoviruses were the most frequently associated viruses. Interestingly, baseline levels of IFNλ1 were lower when compared to healthy controls in this population. During asthma exacerbations, type III IFN in the nasal fluid was determinant for disease severity. Moreover, higher levels of type III Interferons were found associated with wheezing and severity of exacerbation. It was also hypothesized that the lower level of IFNλ1 prior to RV infection could play a role by enhancing susceptibility in patients with asthma (69).

Altogether, these studies point out that we cannot summarize data coming from samples collected with different protocols, as these differences might influence the conclusions. This is demonstrated by the studies mentioned above in which the analysis of the nasal and bronchial fluid lining the airways in adults *in vivo* shows antiviral IFNλ induction in asthma whereas *in vitro* studies in isolated bronchial epithelial cells showing a defect of IFNλ. It becomes clear that there are big differences in adult cohorts and in children. The paediatric studies tend to show a defective induction of IFNλ, which might also be due to the ongoing maturation of the immune system.

#### 3.1.2 Role of IFN Type III During Stable Asthma Condition

In well-controlled asthma patients rhinovirus-induced interferon production was shown not to be impaired. Human bronchial epithelial cells (HBEC) were cultured from asthmatics and healthy subjects and infected with two types of rhinovirus (RV16 and RV1B). Supernatants were analyzed at different time points (8, 24 and 48 hours). HBEC from asthmatic patients produced more IFNλ than IFNβ, indicating that IFNλ was the main IFN subtype found in asthmatic and healthy subjects (70). Neither defective Interferon induction nor an increase in the rhinovirus replication was documented. In addition, a cohort study of control and stable asthmatic adults analyzed IFNλ1 and IFNλ2 expression in sputum samples. IFNλ2 was significantly induced in asthmatic patients while IFNλ1 was not. IFNλ2 was correlated with higher numbers of eosinophils and higher levels of CD3 expression. This could indicate a differential expression or different functions of IFNλ1 and 2 in asthmatic patients (71).

Similarly, IFNλ levels and IFNβ production during rhinovirus infection *in vitro* was not found to be deficient in well controlled asthmatics in an article published by Sykes et al., indicating that in well controlled asthma there is a good IFN type I and III immune response (70).

Furthermore, stable asthmatic patients usually have therapeutically controlled asthma. The treatment of these patients mainly consists of glucocorticoid and ß-sympathomimetic combinational therapy. These therapeutic agents might also have an effect on the IFN mediated host defense. A cohort of 25 atopic asthmatics was pretreated with budesonide, 16 days prior to RV inoculation. The authors didn’t detect differences in the accumulation of RV associated inflammatory cells (72). In contrast, the work by Thomas et al. demonstrates that glucocorticoid treatment can increase the virus replication and suppress the production of type I and III IFNs. In human epithelial cells and a murine model of Influenza-A infection these alterations caused by glucocorticoid pre-treatment could be ameliorated by giving IFNα, IFNβ or IFNλ (73).

### 3.2 Role of IFN Type III in the Peripheral Blood in Asthma

In the airways, IFN-lambda production by epithelial cells might be influenced directly by the virus. By contrast, levels of IFN-lambda measured in the periphery, for example serum or plasma, might reflect some indirect effect of the virus infection in cells of the immune system. They can produce IFNλ but are not necessarily directly infected by the virus. Asthmatic patients generally show a broad variety of cytokine and chemokine production in the peripheral blood. A group of asthmatic children with wheezing during discrete time periods had increased levels of IFNγ, IL-5, IL-4 and IL-10 during exacerbation (74). Especially pDC are circulating in the periphery and are able to produce IFNλ as well as respond to IFNλs binding (75). Therefore, our group investigated type I and type III Interferons in the peripheral blood during baseline and symptomatic visit. In the course of an active RV1B infection in the upper airways, preschool children with and without asthma that showed common cold symptoms were seen as “symptomatic”. At baseline, we found an induction of type III Interferons in serum of asthmatic children positive for RV in the nasal pharyngeal fluid as compared to asthmatic children without RV in the upper airways. Moreover, during symptomatic visit, IFNλ was found consistently upregulated as at this time point all asthmatic children had RV detectable in their airways. We thus concluded that IFNλ producing cells are induced in the periphery in children with asthma and rhinovirus infection. Also these results indicated that there is not a genetic defect of IFNλ production in our asthma cohort of preschool children (63). We next investigated the defect of type I Interferon in the same cohort of preschool healthy and asthmatic children. These IFN members are released classically by plasmacytoid DCs which can be detected in the peripheral blood (76). In this case, we confirmed the defect in IFNα production, at the baseline visit, in asthmatic children, both in PBMCs and in serum. By contrast, during an exacerbation of asthma these children experienced a significant increase of IFNα (63).

IFNλ has also been described to influence the TH1-TH2-balance by suppressing the expression of GATA3 and IL-13 production in peripheral TH2 cells while increasing the production of IFNγ (77). As these alterations in T cells could also affect dendritic cells, another group investigated the secretion of IL-10 and IL-12 in monocyte derived DC. Here IFNλ was given during maturation of mDC and suppressed IL-10 production while inducing IL-12 (55). These data suggest a shift more towards a TH1 immune response due to IFNλ treatment. This is also a possible mechanism how IFNλ is able to improve the allergic asthma exacerbations by suppressing TH2 responses and balancing it more towards TH1 however bearing the risk of increased cytotoxicity.

In conclusion, our group was pioneer in closely investigating the described deficiency in IFN type I and type III in asthma in the peripheral blood of preschool asthmatic children. These findings show that there is not a defect in Type I and Type III interferons during exacerbations of the disease caused by RV, at least in the PREDICTA cohorts we investigated. There is the need to further analyze these findings in different cohorts as several clinical trials have been initiated in which Type I and type III Interferon is used as asthma treatment. Certainly, to challenge IFN-defect in asthma should lead to a better treatment of this disease, especially in children.

### 3.3 Recent Lessons on Type III Interferons During the COVID-19 Pandemic

Alike rhinoviruses, coronaviruses are single-stranded positive-sense RNA viruses. Coronaviruses have been known for a long time as cause for common colds and are also able to cause exacerbations in asthmatic patients. In the current COVID-19 pandemic, many asthmatic patients were afraid of infecting themselves and having a severe disease progression. It is discussed that allergic asthmatics and allergic patients might even show a protective effect. Jackson et al. found a downregulation of SARS-CoV-2 entry receptor ACE2 in patients with high IgE-levels and high allergic sensitization (78). Controversially, patients who suffer from non-TH2-predominant asthma often have comorbidities such as obesity or cardiovascular diseases and might have a higher risk for severe illness (79). According to the latest literature, asthmatic exacerbation rates during the pandemic were reduced compared to numbers of 2019 (80). This effect might be due to the reduction of social contacts, the use of facial masks and the keeping of distance to other people.

Despite these findings a potent defense against infection is especially important for this group of patients. Experiments proved that SARS-CoV-2 is sensitive to IFNβ application. Additionally the infection of NHBE, a human bronchial epithelial cell line, with SARS-CoV-2 led to a comparable strong immune response as to Influenza-A-Virus but to highly differential expressed genes. In this model, SARS-CoV-2 didn´t promote a strong IFN type I and III response. In contrast, the infection led to a substantial activation of inflammatory mediators like IL-6, IL1RA, CCL2, CCL8 CXCL2, CXCL8, CXCL9, and CXCL16 (81). These results suggest an imbalance between the control of the virus replication and a very aggressive inflammatory response upon infection.

Other publications state that the interferon immune response in SARS-CoV infections is delayed compared to Influenza infection (82). A murine model of IFNLR1 KO mice demonstrated that this strain is susceptible to virus infection and shows 10-times higher virus load of SARS-CoV as compared to wild type mice (83). In contrast, an immune profiling analysis of COVID-19 patients showed that IFNλ was associated with an additional inflammatory cluster only found in severe cases (84). In the ongoing infection there are contrary opinions and evidence that interferon levels are associated with higher saliva viral load and also with disease severity (85). Nevertheless, the dominance of inflammatory cytokines in severe COVID-19 cases demonstrates even more the need for a solid interferon response in the early phase of the infection. The type III interferons as well as type I play an important role in the initial stage of disease in controlling the virus replication and therefore a better outcome of the disease.

All things considered, apparent contradictory results have been reported so far regarding the type III Interferon response to viral infection in asthmatic patients. A potential explanation of such mixed results could be the time of analysis, impact of age, the materials used during the study and of course the heterogeneity of asthma as well as the stage of the disease. Yet, we know that IFNλ is a crucial mediator in different immune processes like the antiviral defense, but it is also known for antibacterial and anti-tumoral properties.

### 3.4 Role of IFNλ in the Intestine

The high prevalence of inflammatory bowel disease in the western countries and the rampant increase in its incidence in the developing world, as well as the management of the two major forms i.e. Crohn’s disease and ulcerative colitis, has become a great socio-economic burden. Although the etiopathogensis of inflammatory bowel disease is not completely understood, there is a general consensus that an uncontrolled immune response against commensal flora in genetically predisposed individuals essentially contributes to disease pathogenesis (86). Different cytokines have been identified as key players in the pathogenesis of the auto-inflammatory response with prominent examples including TNF, IL-6, IL-12, IL-17 and interferons. According to the prevailing view, in the context of early acute inflammation stages, IFNα/β can beneficially modulate inflammation by hindering the spread of infection. During the later stages of inflammation, where chronic processes take central stage, the same cytokines amplify disease by fostering the production of pro-inflammatory mediators and intestinal epithelial cell death (87–89). On the other hand, IFNγ aggravates disease by e.g. directly interfering with β-catenin signaling pathways, resulting in lower proliferation and higher apoptosis rates (90). IFNγ also promotes inflammation in experimental colitis by inducing a breakdown of the vascular barrier through disruption of the adherens junction protein VE-cadherin and (91).

Historically, the role of IFNλs in the gut has been addressed predominantly using viral models of gastrointestinal disease in which it played a critical role in controlling early antiviral events at the mucosal barrier thus promoting homeostasis. In contrast to other organs like the lung in which epithelial cells express both the IFNα/β and the IFNλ receptor complex, intestinal epithelial cells predominantly express the IFNλR (83, 92, 93). The role of IFNλ in viral infections of intestinal cells has been addressed by different studies over the past decade. It has been shown that the cross-talk between IFNλ and IL-22 is responsible for restricting rotavirus replication in a STAT1-dependent manner (94). In another study, treatment of established norovirus infection with IFNλ cured mice in a manner requiring non-hematopoietic cell expression of the IFNλR, and was independent of adaptive immunity. Whereas IFNα/β prevented the systemic spread of murine norovirus, only IFNλ was able to control the persistent enteric infection suggesting the therapeutic potential of type III interferons for curing virus infections in the gastrointestinal tract (95). Another study demonstrated that antibiotics and IFNλ prevented persistent murine norovirus infection. IFNλR, as well as the transcription factors STAT1 and IRF3, were required for antibiotics to prevent viral persistence (96). Very recent results indicated a critical role of IFNλ in controlling SARS-CoV-2 infection in human intestinal epithelial cells. The results show that stimulation of human primary intestinal epithelial cells with SARS-CoV-2 resulted in the increased production of IFNλ but not IFNα/β (97). Patankar et al. found that IFNλ stimulation of small intestine organoids derived from wild type mice resulted in a decrease of both SARS-CoV-2 receptors ACE2 and TMPRSS2 expression levels. Moreover, mRNA expression levels of ACE2 were decreased in the small intestine of mice that were injected with an IFNλ expression vector as compared to the empty control vector (98).

Overall, interferons are important at all mucosal sites of entry, regardless if it is the lung or the gut. Because it still remains unclear if asthmatic patients show a defective IFNλ immune response, at least during some periods of their disease, they might also exhibit a different control of infections in the gut. In fact, several studies suggest a lung-gut axis and find more gastrointestinal symptoms in asthmatic children, which might be due to an altered interferon response (99).

## 4 Regulation of Interferon-Lambda Receptor

The effect of Type I and Type III IFN in the airways and the peripheral blood is dependent not only on its stability or production but also on the regulation of its receptor which at the end sequestrates free IFNs. Thus, another variable that must be considered is the regulation of IFN receptors in health and disease. The interferon-lambda receptor (IFNλR) consists of two subunits: the more restricted interferon-lambda receptor 1 (IL-28 Rα) and the ubiquitously expressed IL-10 receptor subunit beta (IL-10Rβ). Interferon-lambda family members binding to their receptor leads to the activation of Janus kinase 1 (JAK1) and Tyrosine kinase 2 (TYK2). This in turn results in the phosphorylation of signal transducer and activator of transcription 1 (STAT1) and STAT2 heterodimers, which afterwards form a complex with IFN regulatory Factor 9 (IRF9). This STAT1-STAT2-IRF9 (ISGF3) complex translocates into the nucleus, inducing several hundreds of IFN-stimulated genes (ISGs). In the literature, the involvement of TYK2 in type III interferon signaling is discussed controversially due to reports that Tyk2 deficiency didn´t abolish IFNλ signaling (100).

IFNλR is expressed more selectively as other IFN receptors. The primary binding of IFNλ happens at the epithelial barrier in the lung and gut on epithelial cells. Immune cells have generally lower levels of IFNλR. B cells, CD4+ and CD8+ T cells express IFNλR while monocytes and NK cells only show low levels of expression (101). Plasmacytoid DC express a basal level of IFNλR that can be upregulated upon activation by viral stimuli (59). Recent publications found responsiveness of neutrophils from the bone marrow, blood and peritoneum to interferon-lambda. IFNλ reduced reactive oxygen species (ROS) production by regulating AKT *via* JAK2 (102). For NK cells there are also reports that find expression of IFNλR in murine and human NK cells or state indirect effects of IFNλ on NK cells (103). The IFNλR KO strain showed higher tumor metastasis in a model of lung cancer. When NK cells where transferred into these mice this effect could be diminished, suggesting the importance of IFNλ in normal NK cell function (104). B cells were also found to carry the IFNλR and were activated by IFNλ and TLR7/8 co-stimulation (105). So far it still remains controversial which immune cells are responding to IFNλ and if that is only the case during pathological processes and infections or also in homeostatic state.

The regulation of the IFNλR plays a central role in controlling resistance against viruses (106). Thus, regulation of the expression of this receptor by both genetic variability and pathogens might influence the functionality of the host defense. Furthermore, biological variation of IFNλR has an impact on the functionality and ability to activate its signaling cascade. This section details recent findings elucidating how the IFNλR expression and function are modulated.

Genetic variation in the form of several single nucleotide polymorphisms (SNPs) in the IFNλ and IFNλR gene region have been described in the literature so far. These SNPs have been associated with a series of important clinical phenotypes in the context of infectious disease (107).

One report found that a SNP in the IFNλR gene led to early treatment failure in HCV patients suggesting the influence of IFNλR on the outcome of therapeutic measures in virus infections (108). Another study showed that eosinophil numbers in the blood were variable due to IFNλR mutations in allergic rhinitis patients. However, no association between SNPs in the IFNλR gene and IgE serum levels was detected (109).

Epigenetic differences between individuals can also be the cause for variations in the expression of IFNλR and the unresponsiveness of cells in the presence of IFNλ. Ding et al. demonstrated that histone deacetylation-mediated closed chromatin conformation and hypermethylation are involved in the silencing of IFNλR expression (110). Methylation of the IFNλR1 gene in human gastric tumor cells and inhibition of methyltransferase lead to increased responsiveness to IFNλ during infection with human norovirus (111).

The detection of a soluble form of the IFNλR (sIFNλR) that compared to the membrane-bound (memIFNλR) form equally strongly binds to IFNλ was surprising. However, this soluble IFNλR fails to respond to its ligation due to the lack of the intracellular signal transduction. This soluble form could indicate a negative feedback regulation. Witte et al. further analyzed the soluble IFNλR and characterized it as a shorter, secreted form of the memIFNλR. It is missing the transmembrane and intracellular domain encoding parts and is equivalent to the extracellular domain of memIFNR except for the five C-terminal amino acids (112). Others hypothesized that there are different splice variants of the IFNλ receptor that have an impact on the potency of its antiviral response (101).

The sIFNλR form is a potential explanation for impaired IFN signaling in some patients. Investigation of this soluble version is still in its infancy, but has the potential to revolutionise our knowledge of IFNλ dependent viral defense. This underlines the need for more research on the soluble form of IFNλR and different splice variants, as well as gene mutations.

## 5 Type III Interferons as Possible Therapeutic Agent

At present, the treatment of asthmatic patients focuses on controlling asthma symptoms and reducing future exacerbations. Glucocorticoids and bronchodilators remain the most prescribed therapy. Last years a novel strategy from GINA (Global Initiative for Asthma) was introduced, recommending a low-dose glucocorticoid and a long-acting beta agonist even in intermittent asthma. The goal is to reduce the chronic inflammation and future exacerbations, as enough data suggests even in mild forms of asthma (113). Subcutaneous immunotherapy in patients with allergic asthma may be beneficial too and should be considered, especially if patients have allergic rhinitis combined with asthma. In contrast, the sublingual immunotherapy has only modest results in asthmatic patients (114).

Type I interferons are already used in therapy up to date. IFNα is a possible treatment option for hepatitis C and B, malignant melanoma and Kaposi-syndrome. IFNβ is a therapeutical approach for multiple sclerosis. Unfortunately, these agents lead to severe side effects like transient changes in peripheral blood or influenza-like symptoms (fever, muscular pain) (115). These IFN type I members are also approved as therapeutic drugs in their PEGylated form. Currently there are also trials of treating COVID-19 patients with IFNα2a that lead to reduced CT scores and accelerated viral clearance (116).

Type III Interferon as a therapeutic agent in asthmatic patients may be of some interest during episodes of exacerbation. In airway epithelial cells, the type III IFN induce the expression of sets of ISGs that are similar to type I IFN, but the kinetic is slightly delayed and has a longer duration in comparison to type I IFN signaling. This could mean a potential clinical use of type III IFNs in preventing excessive antiviral and pro-inflammatory response during viral infections, with only local effects and less generalized damage. As shown in the study conducted by Klinkhammer et al., preventive treatment with IFNλ in mice inhibited the viral replication in the respiratory tract and conferred long-lasting antiviral protection in the upper airways, limiting the transmission to naïve contacts, too (117). Other preclinical studies in mice where intranasal IFNλ was administered demonstrated an improvement in lung inflammation (lower IL-4, IL-5, IL-13 and IL-33) and decrease in eosinophilia (44, 118).

Toll like Receptors (TLRs) recognize pathogen-associated molecular patterns (PAMPs) expressed on infectious agents, like respiratory viruses and induce the development of antiviral and antibacterial immunity. TLRs7 and 8 are located in intracellular compartments of different cell types, predominantly dendritic cells and epithelial cells. Here they detect ssRNA particles from RNA viruses. Upon binding, TLR 7 and 8 lead to activation of the MyD88 signaling pathway *via* IRAK1 (interleukin-1 receptor-associated kinase 1) and IRAK4, resulting in the production of IFN Type I as well as pro-inflammatory cytokines (**Figure 1B**).

Recently, our group found that the toll-like receptor 7 and 8 (TLR7/8) agonist R848 (Resiquimod) induces IFNλR mRNA expression in peripheral blood mononuclear cells (PBMC) of healthy and asthmatic children. By contrast it downregulates the expression of IFNαR1 in PBMC. R848 also induced the levels of immunosuppressive IL-10 and the immunostimulatory cytokine IL-27 (119). These results suggest that the expression of INFλR can be modulated *via* TLR7 and 8. Other working groups hypothesized that SOCS-1 is able to utilize TLR8 to supress TLR7 mediated IFN production in mice (120). Therefore, despite differences in TLR7/8 between human and mouse, the activation of TLRs is not necessarily positive for infection control and immune defense.

Conversely, to the production of pro-inflammatory mediators, experimental studies have revealed a protective effect of R848 in experimental asthma. The numbers of cells in the bronchoalveolar lavage, as well as IgE, IL-4 and IL-5 levels were reduced in mice treated with Resiquimod before challenge (121–125). Grela´s group demonstrated that some of these effects can be transferred by treating invariant NKT cells with R848, and injecting them intra veniously to sensitized mice before challenging them (124). Furthermore, Jha et al. tested an R848-containing nasal spray in healthy, allergic, and allergic asthmatic patients. They discovered higher levels of IFNα, as well as pro-inflammatory cytokines in the nasal mucosal fluid after treatment in both allergic groups compared to healthy controls. A systemic activation of the immune response was not observed. The R848 treatment of the patients in this cohort was very well tolerated and helped to induce a better antiviral immune response (126). A report also stated that R848, in synergy with a TLR3 agonist, activates the production of type I and III interferons by monocyte derived dendritic cells (125).

In summary, these findings reveal TLR7/8 agonists as a potential target to modify the IFN type I and type III dependent immune responses (**Table 1**). As some cell types are not responsive to IFNλ alone it might be a possible co-treatment option to induce IFNλR expression by giving TLR7/8 agonists. R848 is even a potential candidate as adjuvant used in vaccinations. There are already promising results in influenza vaccines that will be explored further in the future (127, 128).

**Table 1:** Overview of important literature on the use of resiquimod (R848) as therapeutic agent in animal models of asthma.

References	Model	Subject	Outcome
Camateros et al. (121)	R848 was applied i.p 24h before each OVA challenge	8-10 weeks old brown Norway rats	R848 reduced IgE, IL-4, IL-5 and BAL cell numbers
Jirmo et al. (122)	R848 i.n one day before OVA challenge +/- anti-IL27 pretreatment	6-8 weeks old C%&BI/6 mice	R848 reduced IgE, AHR and cytokine production, R848 mediated effects are IL-27 dependent
Van et al. (123)	R848 1 hour i.p before 2 of 4 OVA challenges	8-9 weeks old C57BI/6 o NOD mice	R848 increased CD25+ and Foxp3+ T cell numbers *via* TGF β, CD25 depletion attenuated R848 effects
Grela et al. (124)	Transfer of NKT cells from R848 pre-treated OVA challenged mice	6-8 weeks old C57BI/6 or IFNγ deficient mice	R848 pre-treated NKT cell transfer reduces AHR and eosinophil numbers, NKT cell dependent effects are IFNγ driven

Taken together, we need to improve the understanding of the interactions between viral infections and Type III Interferons, in order to prevent and treat exacerbations in asthma successfully. The emerging type III IFN therapies are promising and can lead to an individual and more specific treatment of exacerbations.

## 6 Outlook

Reflecting on the discovery of interferons over the last 20 years has majorly advanced our understanding of virus resistance. As virus infection can induce exacerbations in asthmatics associated with increased disease severity, this review focuses on the mechanisms of interferon immune response in asthmatic patients, especially children. The literature on this topic remains very inconsistent and the methods employed do not always allow direct comparison of results. So far it hasn´t been proven that asthmatics have an impaired interferon response. However, there is emerging evidence that their viral clearance differs from that of healthy subjects. Some SNPs also suggest a connection between genetic mutations in the interferon-lambda gene and the development of autoimmune diseases. Modulation of the IFNλR *via* TLR7 and 8 agonists provides a means of interfering with the viral defense process. Moreover, the finding of a soluble IFNλR form raises new questions in this field that need to be investigated in greater depth.

The COVID-19 pandemic is a harsh reminder of the necessity of a solid viral defense line. A recent phase II clinical trial highlights the therapeutic potential of IFNλ response, using Peginterferon-lambda to treat COVID-19 patients, based on the assumption that interferons are the first combatants in the clearance of SARS-CoV-2, not only in the lung, but also in the whole organism (129). Nevertheless, we are only beginning to understand the possibilities inherent in altering the IFNλ response and the potential impact on our patients.

## Author Contributions

SK and CS wrote the manuscript, designed the figures and reviewed the current literature. SK and CS contributed equally to the manuscript and thus share the first authorship. JG wrote the paragraph on rhinovirus infection and antiviral immune response and created **Figure 1A**. SZ helped by providing clinical expertise on the literature. MC helped by providing expertise on the literature of IFNλ in the gut. SF supervised the work and edited the manuscript for submission. All authors contributed to the article and approved the submitted version.

## Funding

SK is supported by the Interdisciplinary Center for Clinical Research (IZKF) at the University Hospital of the University of Erlangen-Nuremberg (Project A82). This work was supported by a grant, awarded to SF, from the Collaborative Research Centre (CRC) 1181 for the project TP-B08 N (Molecular mechanisms controlling regulatory T cell activation in the resolution of asthma), at the University hospital in Erlangen, Germany.

## Conflict of Interest

The authors declare that the research was conducted in the absence of any commercial or financial relationships that could be construed as a potential conflict of interest.

## Publisher’s Note

All claims expressed in this article are solely those of the authors and do not necessarily represent those of their affiliated organizations, or those of the publisher, the editors and the reviewers. Any product that may be evaluated in this article, or claim that may be made by its manufacturer, is not guaranteed or endorsed by the publisher.
